# Differential Expression of Cytokines in Response to Respiratory Syncytial Virus Infection of Calves with High or Low Circulating 25-Hydroxyvitamin D_3_

**DOI:** 10.1371/journal.pone.0033074

**Published:** 2012-03-08

**Authors:** Randy E. Sacco, Brian J. Nonnecke, Mitchell V. Palmer, W. Ray Waters, John D. Lippolis, Timothy A. Reinhardt

**Affiliations:** 1 Ruminant Diseases and Immunology Research Unit, National Animal Disease Center, United States Department of Agriculture, Agricultural Research Service, Ames, Iowa, United States of America; 2 Infectious Bacterial Diseases Research Unit, National Animal Disease Center, United States Department of Agriculture, Agricultural Research Service, Ames, Iowa, United States of America; University of Georgia, United States of America

## Abstract

Deficiency of serum levels of 25-hydroxyvitamin D_3_ has been related to increased risk of lower respiratory tract infections in children. Respiratory syncytial virus (RSV) is a leading cause of low respiratory tract infections in infants and young children. The neonatal calf model of RSV infection shares many features in common with RSV infection in infants and children. In the present study, we hypothesized that calves with low circulating levels of 25-hydroxyvitamin D_3_ (25(OH)D_3_) would be more susceptible to RSV infection than calves with high circulating levels of 25(OH)D_3_. Calves were fed milk replacer diets with different levels of vitamin D for a 10 wk period to establish two treatment groups, one with high (177 ng/ml) and one with low (32.5 ng/ml) circulating 25(OH)D_3_. Animals were experimentally infected via aerosol challenge with RSV. Data on circulating 25(OH)D_3_ levels showed that high and low concentrations of 25(OH)D_3_ were maintained during infection. At necropsy, lung lesions due to RSV were similar in the two vitamin D treatment groups. We show for the first time that RSV infection activates the vitamin D intracrine pathway in the inflamed lung. Importantly, however, we observed that cytokines frequently inhibited by this pathway *in vitro* are, in fact, either significantly upregulated (IL-12p40) or unaffected (IFN-γ) in the lungs of RSV-infected calves with high circulating levels of 25(OH)D_3_. Our data indicate that while vitamin D does have an immunomodulatory role during RSV infection, there was no significant impact on pathogenesis during the early phases of RSV infection. Further examination of the potential effects of vitamin D status on RSV disease resolution will require longer-term studies with immunologically sufficient and deficient vitamin D levels.

## Introduction

1,25-dihydroxyvitamin D_3_ (1,25(OH)_2_D_3_) has long been known to be a primary regulator of calcium homeostasis and bone remodeling [1]. However, recent evidence suggests additional roles for vitamin D in hematopoietic cell differentiation and immune function [2], [3]. It is known that the nuclear vitamin D receptor (VDR) and the enzymes responsible for activation (CYP27B1; 1α-hydroxylase) and degradation (CYP24A1; 24-hydroxylase) of vitamin D are expressed in subsets of immune cells. Moveover, several aspects of immune regulation are modified by the actions of vitamin D [2], [4], [5], [6], [7]. In fact, vitamin D-responsive elements have been identified in the promoter region of a number of genes with a role in immunity [8]. An examination of literature from the last three decades of research shows that equivocal results have been observed regarding the effect of vitamin D on pro-inflammatory cytokine expression. *In vitro* studies have shown that proinflammatory cytokine expression is increased by 1,25(OH)_2_D_3_ treatment [9], [10], [11] or is downregulated [12], [13]. In addition, vitamin D appears to suppress the generation of T helper 1 (T_H1_) immune responses, known to be critical for clearance of many bacterial and viral infections [14], [15], [16].

Respiratory infections remain a major cause of morbidity and mortality in children world-wide [17]. Recent epidemiological studies suggest a connection between inadequate vitamin D concentrations and respiratory tract infections in children. Available data indicate an association between low vitamin D status and tuberculosis in children [18]. Moreover, deficiencies in vitamin D have been associated with an increased incidence of lower respiratory tract infections [19], [20], [21], of which respiratory syncytial virus (RSV) is the leading cause in infants and children worldwide [22]. In addition, the incidence of viral infections in the pediatric population frequently peaks in winter months when cutaneous vitamin D synthesis is at its lowest level [22], [23]. Taken together, these observations suggest a potential role for vitamin D in modulating the immune response to respiratory infections in the young host.

Based on available epidemiological evidence and the potential role for vitamin D in immune regulation, studies have been initiated to examine the ability of vitamin D to modulate the response to *in vitro* respiratory infections. Early studies indicate *in vitro* 1,25(OH)_2_D_3_ treatment of IFN-γ-activated human monocytes enhanced the ability of these cells to inhibit *Mycobacterium tuberculosis* [24], [25]. In a later pivotal paper, Liu et al. [26] demonstrated a mechanism for the inhibition of *M. tuberculosis* growth by vitamin D treatment. Pathogen activation of monocytes induces 1α-hydroxylase (1α-OHase), promoting conversion of 25-hydroxyvitamin D_3_ (25(OH)D_3_) to the active metabolite 1,25(OH)_2_D_3_. This study further found that 1,25(OH)_2_D_3_ induces cathelicidin expression, which in turn inhibits the growth of *M. tuberculosis* in monocyte cultures. Recently, it was shown that treatment of cultured respiratory epithelial cells with 1,25(OH)_2_D_3_ decreases RSV induction of pro-inflammatory gene expression [27]. In spite of the reduction of antiviral IFN-β, there was no concomitant increase in RSV replication, suggesting that providing adequate vitamin D could reduce inflammation while maintaining antiviral activity. Although these studies suggest that vitamin D supplementation may be beneficial to individuals with respiratory illnesses, two recent double-blind, placebo controlled trials in TB patients report conflicting results [28], [29]. In a recent review, Bruce et al. [30] suggests that data from several models of infection do not provide sufficient evidence to support a role for vitamin D in affecting the course of disease. However, it has been recently shown in the lactating dairy cow that 25(OH)D_3_ administration can reduce the severity of an intramammary infection [31]. Thus, it is clear that there is a need for additional *in vivo* studies to examine the potential role of vitamin D in modulating experimental viral respiratory infections.

We examined the influence of vitamin D status on the response to RSV experimental challenge in calves. Calves with high or low circulating 25(OH)D_3_ levels were challenged with RSV and subsequently, lung tissue samples examined at day 7 postinfection. We show, for the first time *in vivo*, that RSV infection induced expression of the VDR and associated hydroxylase enzymes in the lung. Importantly, gene expression levels of pro-inflammatory cytokines were not suppressed in the presence of this induced vitamin D regulatory network in the lung, but rather specific pro-inflammatory cytokines were elevated in the high vitamin D group compared to the low vitamin D group of calves.

## Results

### Serum 25(OH)D_3_ concentrations

**Figure 1:**
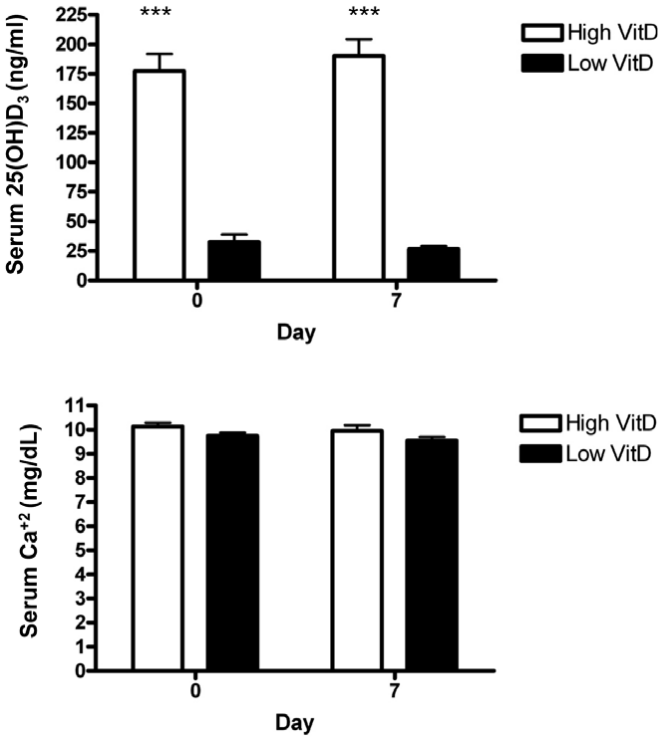
Serum 25(OH)D3 and Ca^+2^ concentrations in calves at the beginning and the termination of the bovine RSVexperimental infection. Calves (n = 8 per treatment group) were maintained on diets containing 1700 IU of vitamin D/kg (low vitD) or 17,900 IU of vitamin D/kg (high vitD) for ten weeks prior to experimental challenge. Calves were challenged via aerosol inoculation with bovine RSV. Blood samples were collected from each calf on the day of experimental challenge and just prior to euthanasia. (A) Serum 25(OH)D_3_ was quantified by radioimmunoassay and data shown represent the mean ± SE. Data were analyzed using Student's t-test. ***P<.001. (B) Serum calcium was determined by atomic absorption spectrometry and data shown represent the mean ± SE.

We sought to examine the influence of vitamin D levels on the response to RSV experimental challenge. Over a ten week period, we were able to establish two groups of calves with differing levels of circulating 25(OH)D_3_. Data in Fig. 1A shows levels of serum 25(OH)D_3_ at the time of infection (day 0) and at the completion of the experiments (day 7). Calves in the high vitamin D group had significantly (P<.001) higher circulating levels of 25(OH)D_3_ at the initiation of challenge compared to the low vitamin D group (177.3 ng/ml versus 32.5 ng/ml). The difference in 25(OH)D_3_ between the treatment groups was maintained over the course of the experimental infection period with circulating levels of 189.9 ng/ml and 26.5 ng/ml for the high vitamin D group and low vitamin D groups at day 7 postinfection, respectively. There were no significant differences in serum Ca^+2^ (Fig. 1B), phosphorus, or Mg^+2^ (data not shown for phosphorus or Mg^+2^) between the two vitamin D treatment groups. These latter data indicate that calcium homeostasis was not affected by differences in vitamin D supplementation.

### Clinical signs

Rectal temperatures were recorded daily for calves in each treatment group. There were no significant differences in body temperature between the high vitamin D and the low vitamin D groups (data not shown). In addition, none of the calves exhibited a substantially prolonged elevation in body temperature following RSV infection. Moreover, only mild coughs of short duration were observed in the high vitamin D treatment group, with no coughing observed in the low vitamin D treatment group. Two calves in the high vitamin D group had increased respiration rates and reduced feed consumption of <24 hr duration.

### Gross lesions

**Figure 2:**
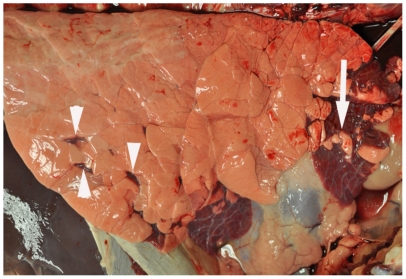
Right lung from an RSV-infected calf at day 7 post-infection. Note multifocal to coalescing areas of plum-red consolidation in cranial and ventral aspects of right cranial and middle lobes. Consolidated areas surround and divide foci of pale pink hyperinflated lung (arrow). Scattered multifocal areas of consolidation are also present in the ventral third of the right caudal lobe (arrowheads).

Gross lesions consisted of bilateral, multifocal, firm, plum-red areas of consolidation that were of variable size and depressed compared to the adjacent normal appearing lung (Fig. 2). Lesions were more frequently observed in cranioventral lung lobes. On cut surface, areas of consolidation were well delineated from adjacent normal lung. In some cases, areas of consolidation surrounded and divided regions of pink, hyperinflated lung. No differences in gross lesion severity were noted between high vitamin D and low vitamin D status calves.

### Histopathological lesions

**Figure 3:**
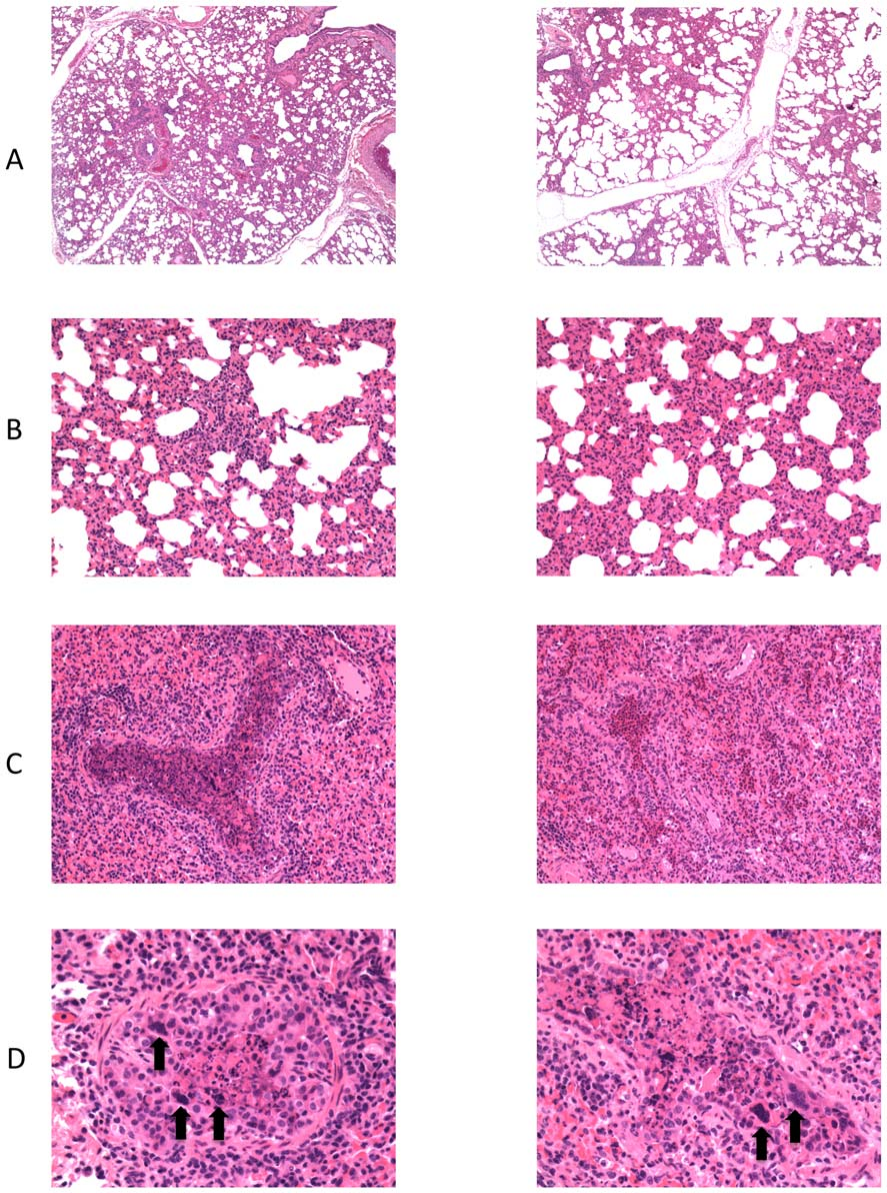
Histological lesions observed in lung of high and low vitamin D treatment groups of calves after experimental infection with bovine RSV. Calves with low or high circulating 25(OH)D_3_ were challenged via aerosol with bovine RSV. On day 7 post-infection, samples of lung were collected for histological evaluation. A representative image from a single calf for each treatment group (low vitD left panels; high vitD right panels) is shown. (**A**) Moderate interstitial thickening and wide interlobular septae due to edema. Original magnification, 4×. (**B**) Alveolar septae are thickened due to cellular infiltrates found to be macrophages, lymphocytes and lesser numbers of neutrophils when viewed at higher magnification. Original magnification, 20×. (**C**) Bronchioles are filled with neutrophils, sloughed epithelial cells, and necrotic cell debris. Original magnification, 20×. (**D**) There is partial to complete loss of bronchiolar epithelial cells with attenuation of remaining cells. In some bronchioles, epithelial cells form multinucleated syncytial cells (arrows). Original magnification, 40×.

Significant microscopic lesions are shown with a representative photomicrograph from the low vitamin D group in the left panels and a representative photomicrograph from the high vitamin D group in the right panels (Fig. 3A–D). It should be noted that average histological lesion scores were similar in the two groups. The high vitamin D status group had an average lesion score of 3.5, while the low vitamin D status group had an average lesion score of 3.9. Microscopically, interlobular septa were expanded by clear space interpreted to be edema (Fig. 3A). Alveolar septa were thickened due to infiltrates of macrophages, lymphocytes, and lesser numbers of neutrophils (Fig. 3B). Intralesional bronchioles were filled with neutrophils, sloughed epithelial cells, and necrotic cellular debris (Fig. 3C). Bronchiolar epithelial cell necrosis resulted in attenuation of remaining epithelial cells, or complete loss of airway epithelium. In some bronchioles, epithelial cells formed multinucleated syncytial cells (Fig. 3D).

### Virus isolation

Nasal swabs and frozen lung tissue samples were collected for virus isolation on the day of necropsy. In experiment 1, virus was re-isolated from all calves in the high and low vitamin D status groups. In experiment 2, virus was isolated from 7 out of 8 calves, with the exception being that virus was not re-isolated from one of the low vitamin D status group calves. Thus, high vitamin D status did not provide an apparent advantage in regards to RSV clearance.

### VDR and hydroxylase gene expression in lung tissues

**Figure 4:**
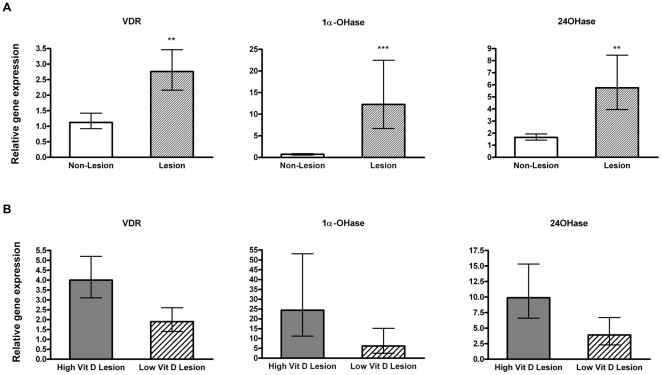
Nuclear VDR and hydroxylase gene expression levels in lung tissues of calves following bovine RSV experimental infection. (**A**) Vitamin D receptor (VDR), CYP27B1 (1α-OHase), or CYP24A1 (24-OHase) mRNA levels in lesioned or non-lesioned lung samples from bovine RSV-infected calves irrespective of vitamin D treatment (n = 16). (**B**) Vitamin D receptor (VDR), CYP27B1 (1α-OHase), or CYP24A1 (24-OHase) mRNA levels in lesioned lung samples from bovine RSV-infected calves with low (n = 8) or high (n = 8) circulating 25(OH)D_3_ levels. The amount of each target gene was determined by quantitative real-time PCR with normalization to RPS9. ΔΔCt values ± SE were transformed (2^−ΔΔCt^) and data are presented as the target gene expression relative to control lung samples (n = 4). Statistical analysis was performed using Student's t-test. ΔΔCt values were used in the analysis of relative gene expression. **P<.01,***P<.001.

The effects of vitamin D on modulation of downstream genes expressing vitamin D response elements (VDREs) depend on the presence of the VDR. Therefore, gene expression of the VDR, 1α-OHase, and 24-OHase was measured in the lungs of RSV-infected calves with differing serum levels of 25(OH)D_3_. VDR was expressed at significantly (P<.01) higher levels in sections of lesioned lung tissue compared to non-lesioned tissue across vitamin D treatment groups (Fig. 4A). Moreover, both 1α-OHase and 24-OHase were strongly upregulated (P<.001) in RSV-induced lung lesions. There was a similar gene expression of the VDR, 1α-OHase, and 24-OHase in the non-lesioned lung samples relative to non-infected control lung samples. Although the high vitamin D group tended to have higher VDR gene expression (p = 0.09), 1α-OHase, and 24-OHase, there was no significant influence of circulating serum 25(OH)D_3_ levels on the expression of these three genes in lesioned lungs (Fig. 4B). These data indicate that vitamin D intracrine regulatory pathways in the lung are activated by RSV.

### Pro-inflammatory cytokine gene expression levels in lung tissues

**Figure 5:**
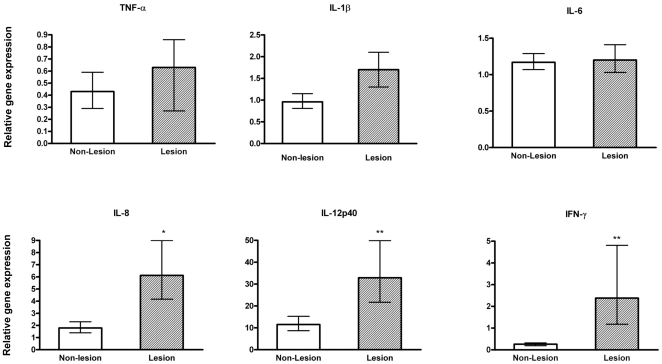
Pro-inflammatory cytokine gene expression levels in lung tissues of calves following bovine RSV experimental infection. Inflammatory cytokine RNA levels in lesioned or non-lesioned lung samples from bovine RSV-infected calves irrespective of vitamin D treatment (n = 16). The amount of each target gene was determined by quantitative real-time PCR with normalization to RPS9. ΔΔCt values ± SE were transformed (2^−ΔΔCt^) and data are presented as the expression relative to control lung samples (n = 4). Statistical analysis was performed using Student's t-test. ΔΔCt values were used in the analysis of relative gene expression. *P<.05, **P<.01.

**Figure 6:**
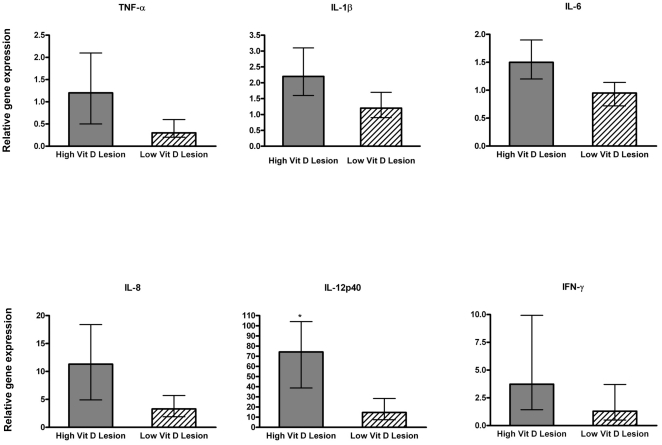
Pro-inflammatory cytokine gene expression levels in lesioned lung tissues of calves with high or low circulating 25(OH)D_3_ following bovine RSV experimental infection. Inflammatory cytokine mRNA levels in lesioned lung samples from bovine RSV-infected calves with low (n = 8) or high (n = 8) circulating 25(OH)D_3_ levels. The amount of each target gene was determined by quantitative real-time PCR with normalization to RPS9. ΔΔCt values ± SE were transformed (2^−ΔΔCt^) and data are presented as the target gene expression relative to control lung samples (n = 4). Statistical analysis was performed using Student's t-test. ΔΔCt values were used in the analysis of relative gene expression. *P<.05.

RSV infection induces a wide range of inflammatory mediators in antigen-presenting cells and respiratory epithelial cells [32], [33], [34], [35], [36], [37] and *in vitro* studies have suggested that vitamin D may suppress these cytokines [38], [39], [40]. Based on this information, it was intriguing to speculate that animals provided high dietary levels of vitamin D would have reduced pro-inflammatory cytokines in response to RSV infection compared to animals provided low dietary levels of vitamin D. As would be predicted, RSV infection induced elevated levels of inflammatory cytokines, including significant IL-8, IL-12p40, and IFN-γ mRNA levels in lung lesions of RSV-infected calves compared to levels in non-lesioned lung across vitamin D treatment groups (Fig. 5). Further, it is of note that IL-12p40 mRNA levels were 11-fold higher in non-lesioned lung samples from RSV-infected calves relative to levels in control lung tissue. Interestingly, IL-12p40 was significantly elevated in calves with high serum levels of 25(OH)D_3_ (Fig. 6), and contrary to *in vitro* studies, neither the activation of local 1,25(OH)_2_D_3_ nor high vitamin D status resulted in inhibition of IFN-γ. Moreover, there was a trend (p = 0.1) for elevated levels of pro-inflammatory IL-8 in the high vitamin D treatment group. Thus, during experimental *in vivo* RSV infection vitamin D supplementation does not suppress the pro-inflammatory cytokine response in the inflamed lung at 7 d postinfection.

### Anti-inflammatory cytokine gene levels in lung tissues

**Figure 7:**
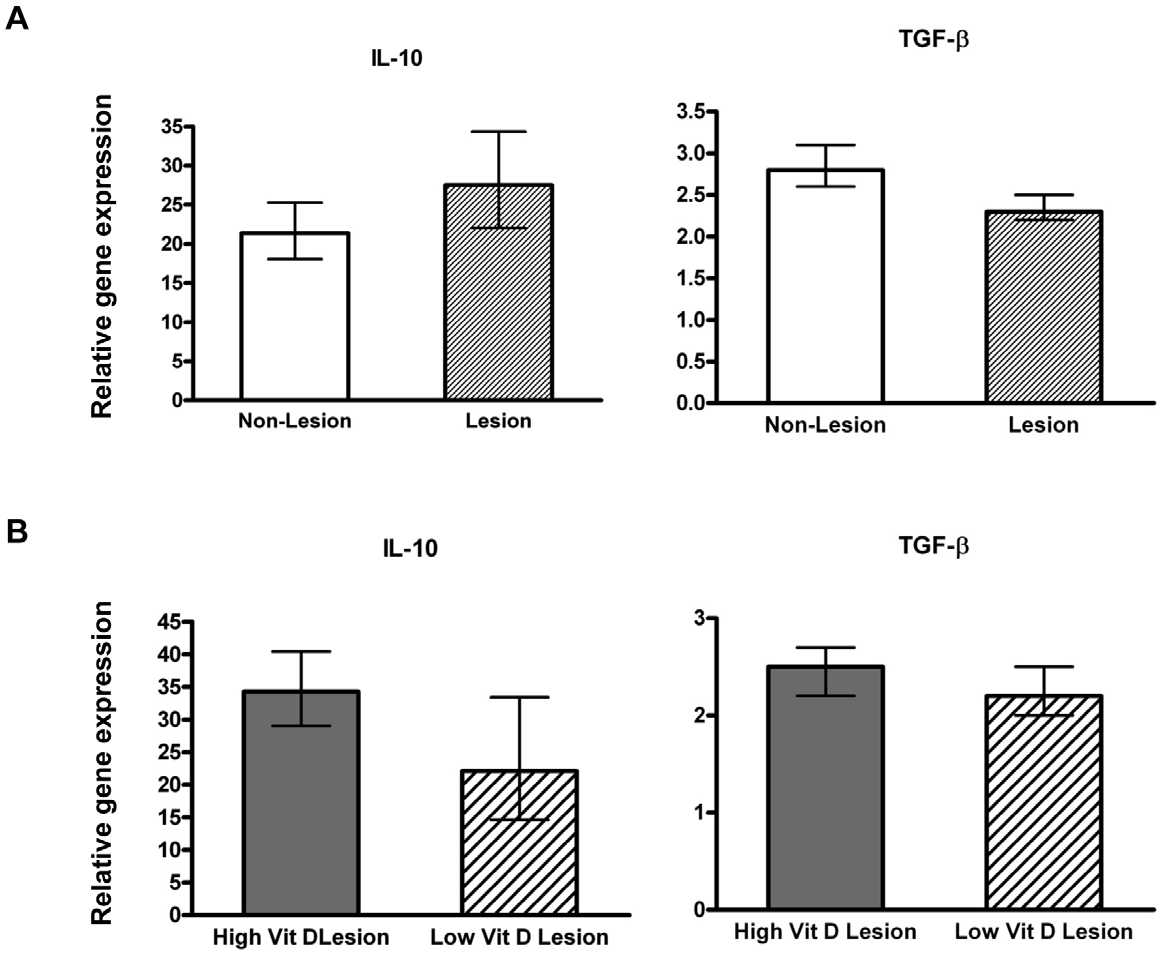
Anti-inflammatory cytokine gene expression levels in lung tissues of calves following bovine RSV experimental infection. (**A**) Anti-inflammatory cytokine mRNA levels in lesioned or non-lesioned lung samples from bovine RSV-infected calves irrespective of vitamin D treatment (n = 16). (**B**) Anti-inflammatory cytokine mRNA levels in lesioned lung samples from bovine RSV-infected calves with low (n = 8) or high (n = 8) circulating 25(OH)D_3_ levels. The amount of each target gene was determined by quantitative real-time PCR with normalization to RPS9. ΔΔCt values ± SE were transformed (2^−ΔΔCt^) and data are presented as the target gene expression relative to control lung samples (n = 4). Statistical analysis was performed using Student's t-test. ΔΔCt values were used in the analysis of relative gene expression.

TGF-β and IL-10 are known to have pronounced immunomodulatory and immunosuppressive function, which can be critical in limiting tissue damage associated with immune reactivity to pathogens at environmental interfaces [41]. In this study, we found IL-10 gene expression was elevated more than 25-fold in both lesioned and non-lesioned sections of RSV-infected lungs compared to control lung tissue (Fig. 7). TGF-β gene expression tended to be higher in non-inflamed lung tissue sections compared to inflamed lung tissue sections. In lesioned lung sections of RSV-infected calves, IL-4 was not consistently detected (data not shown). Further, we did not observe any significant differences in IL-10 or TGF-β gene expression between vitamin D treatment groups in response to RSV infection. Our data show an induction of cytokines aimed at reducing tissue inflammation in response to RSV infection that is not dependent on vitamin D status.

### Expression of SOCS1 and SOCS3 in lung tissues

**Figure 8:**
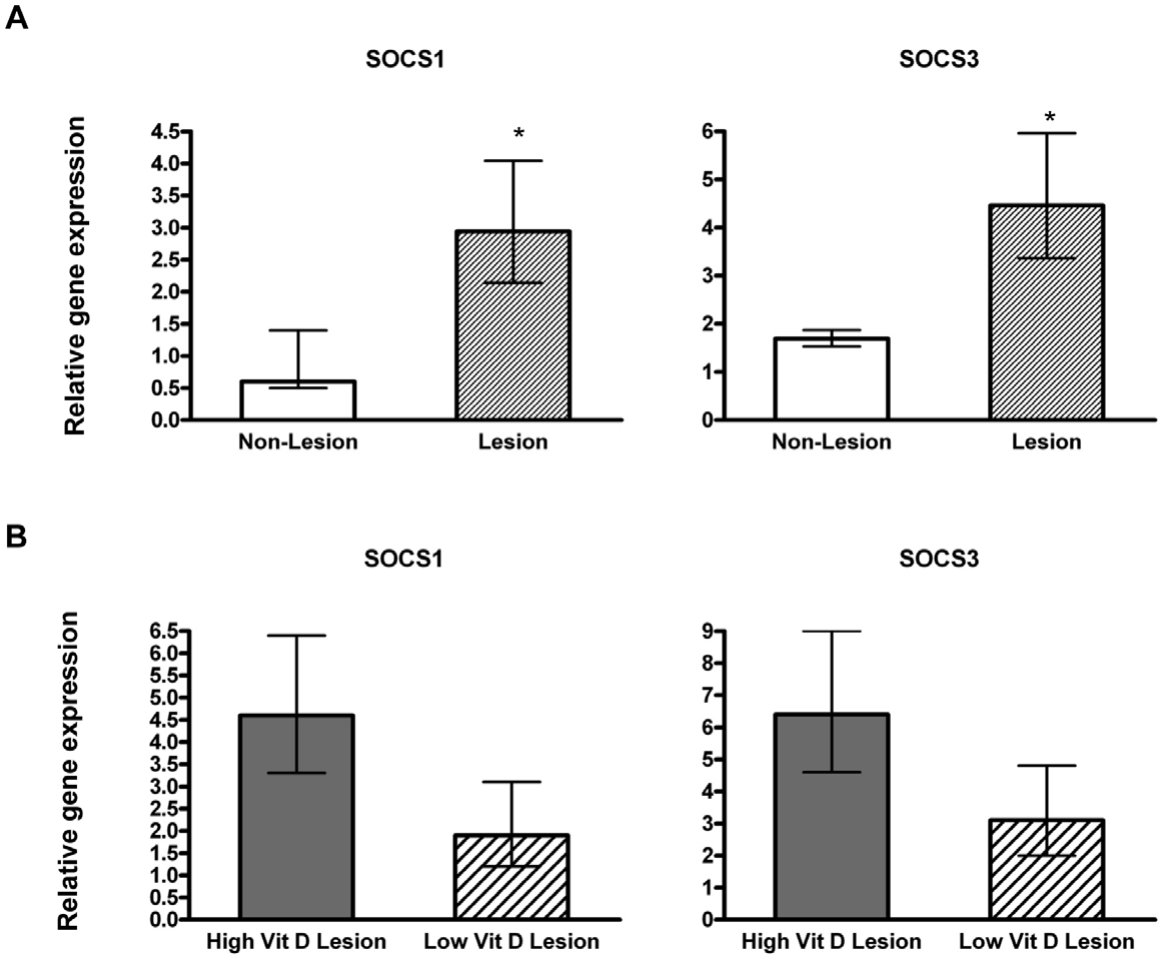
SOCS1 and SOCS3 gene expression in lung tissues of calves following bovine RSV experimental infection. (**A**) Suppressors of cytokine signaling (SOCS) 1 or SOCS3 mRNA levels in lesioned or non-lesioned lung samples from bovine RSV-infected calves irrespective of vitamin D treatment (n = 16). (**B**) Suppressors of cytokine signaling (SOCS) 1 or SOCS3 mRNA levels in lesioned lung samples from bovine RSV-infected calves with low (n = 8) or high (n = 8) circulating 25(OH)D_3_ levels. The amount of each target gene was determined by quantitative real-time PCR with normalization to RPS9. The relative amount of each target gene was determined using the 2^−ΔΔCt^ method. ΔΔCt values ± SE were transformed (2^−ΔΔCt^) and data are presented as the target gene expression relative to control lung samples (n = 4). Statistical analysis was performed using Student's t-test. ΔΔCt values were used in the analysis of relative gene expression. *P<.05.

RSV attachment (G) and nonstructural proteins (NS) have been shown to modulate the expression of SOCS1 and SOC3 in a mouse lung epithelial cell line [42]. On the other hand, there is very limited data on the influence of vitamin D on SOCS expression. Therefore, we examined lung expression of SOCS1 and 3 in response to RSV infection of calves with differing circulating vitamin D levels (Fig. 8). SOCS1 and SOCS3 were expressed at significantly higher levels in lesioned versus non-lesioned lung samples from RSV-infected calves of both vitamin D treatment groups relative to levels in control lung samples. However, we did not observe significant differences in their expression based on vitamin D treatment, even though there was a tendency for higher SOCS expression in the high vitamin D group.

## Discussion

There has been much recent attention given to the role of vitamin D in regulating host immune responses and by extension, to the role that it might play in host resistance to infection. Epidemiological studies have suggested an association between low vitamin D levels and respiratory infections [19], [20]. Recently, vitamin D was found to dampen *in vitro* inflammatory responses of pulmonary epithelial cells to RSV infection without a concomitant increase in viral replication [27]. Thus, it is of interest to determine whether vitamin D supplementation could be effective against an *in vivo* RSV infection. In this study, we have shown that pro-inflammatory cytokine responses to respiratory syncytial virus infection are, in fact, elevated in the lungs of calves receiving high levels of dietary vitamin D supplementation compared to those receiving low levels of dietary vitamin D. However, in spite of the higher levels of pro-inflammatory cytokines, we did not observe any differences in severity of lung lesions between the vitamin D treatment groups at a time point chosen when there is significant lung involvement in ruminant models of RSV infection [43], [44].

We believe that there are several major advantages to the bovine model system employed in the present study for examining the influences of vitamin D supplementation on RSV infection. Naturally occurring RSV infection in young ruminants mimics pathogenesis and lesions observed in RSV infection of infants and children [43], [45], [46], [47]. As is the case for humans, neonatal calves and lambs are more susceptible to RSV than older animals. In addition, alveolar development begins prenatally in humans and ruminants, whereas mice have postnatal alveolar development [48], [49]. Finally, calves provide a valuable model for estimating the vitamin D requirements for an adequately functioning human immune system. A concern of the mouse model is that the vitamin D requirements of the mouse differ from those of humans and cattle. At least a partial explanation for these differences is likely due to the fact mice are nocturnal and do not rely on endogenous synthesis of vitamin D_3_ in the skin to assure vitamin D adequacy [26].

Our data showing that established differences in circulating 25(OH)D_3_ levels did not alter gross or microscopic lesions induced by RSV infection adds to the conflicting body of literature on the potential for vitamin D to modify the outcome of an experimental infection. There are several previous studies that have examined the relationship between vitamin D and experimental infection by utilizing mouse models with targeted disruptions of vitamin D signaling pathways or providing vitamin D supplementation. In response to *Listeria moncytogenes* infection, VDR^−/−^ mice were able to clear a primary or secondary infection, although kinetics was delayed in the absence of the VDR [50]. Similarly, VDR knockout mice showed normal clearance of *Leishmania major* [51]. By contrast, vitamin D-deficient mice were more susceptible to *Mycobacterium bovis* infection compared to vitamin D-sufficient mice [52]. Treatment of mice with 1,25(OH)_2_D_3_ did not alter clearance of *Candida albicans* or herpes simplex virus [53]. On the other hand, we have recently shown that treatment with 25(OH)D_3_ reduced the severity of an acute bacterial infection in the bovine mammary gland [31]. It is plausible that the role of vitamin D in modulating the response to infection will differ depending on the nature of the inciting pathogen as well as which functional division of the immune system is primarily responsible for disease resolution.

Vitamin D is likely to have complex effects on pulmonary cell biology and lung immunity. It is known that respiratory epithelial cells express high baseline levels of activating 1α-OHase and low levels of catabolizing 24-OHase [54]. We provide evidence that extarenal induction of 1α-OHase exists in the lung during experimental RSV infection. Extrarenal production of 1α-OHase may be stimulated by cytokines such as IL-1 and IFN-γ [55], [56], cytokines we show are upregulated in lung lesions of RSV-infected calves. Upregulation of genes involved in vitamin D regulation in the infected lung has major implications because it allows for local control of 1,25(OH)_2_D_3_ synthesis. In addition, induction of 1α-OHase and VDR expression would subsequently enhance the expression of multiple downstream genes containing VDREs in their promoter region [57].

There exist similarities between cattle and humans in regards to vitamin D metabolism and immune function, such that cattle should be considered a useful model for examination of vitamin D requirements for proper immune function in humans. The substrate for 1α-OHase is 25(OH)D_3_, so the production of bioactive vitamin D depends on the availability of its substrate. Circulating concentrations of 25(OH)D_3_ depend on exposure to sunlight and dietary intake of vitamin D [58]. 25(OH)D_3_ serum levels in cattle typically range from 20 to 50 ng/ml [59]. In the present study, circulating 25(OH)D_3_ levels (32.5 ng/ml) in the low vitamin D group at the initiation of the RSV challenge experiment were above levels (20 ng/ml) considered adequate for calcium homeostasis and levels (30 ng/ml) currently suggested to be sufficient for proper immune function in humans [58]. However, at day 7 following RSV infection, serum levels of 25(OH)D_3_ were slightly below 30 ng/ml in the low vitamin D group. In spite of the differing levels of 25(OH)D_3_, lung lesions induced by RSV infection in calves were similar in the two vitamin D treatment groups. It is unclear at present whether the higher induction of pro-inflammatory cytokines would be protective or detrimental in resolution of RSV infection in the high vitamin D treatment group, since in this study we did not examine later time points of disease resolution.

Previously, we and others have shown that RSV infection induces inflammatory cytokines in antigen-presenting cells and respiratory epithelial cells, the kinetics of which differs between cytokines [32], [33], [34], [35], [36], [37]. For example, we found peak induction of IL-1β mRNA in antigen-presenting cells occurs on day 3 post-infection in the neonatal ruminant RSV model [36]. In the present study, we show a significant upregulation of the pro-inflammatory cytokine IL-8 in lesioned lungs of RSV-infected calves on day 7 postinfection. Importantly, our results fit well with data showing that IL-8 is elevated in the respiratory tract of children with RSV brochiolitis [60], [61]. Rodents lack a *bona fide* homologue of IL-8, although mice have what are considered to be functional homologues, CXCL1 (GRO/KC), CXCL2 (MIP-2) and CXCL5-6 (LIX), that belong to the same major cluster of chemokines [62]. It is therefore evident that the neonatal calf model can prove useful as an *in vivo* platform for future exploration into pathways that specifically regulate IL-8 responses to RSV infection.

Critical to the induction of inflammatory cytokines by the innate immune system are pattern recognition receptors (PRRs) that recognize evolutionarily conserved pathogen-associated molecular patterns, such as nucleic acids. Recognition of viral nucleic acids involves two distinct PRRs, retinoic acid inducible gene-I (RIG-I)-like RNA helicases and toll-like receptors (TLRs). Among multiple TLR isoforms expressed in airway epithelial cells, TLR3 is one of the most abundant [63]. It has been shown that RSV induces cytokine/chemokine production in airway epithelial cells via signaling through TLR3 and RIG-I, which are linked to distinct pathways controlling NF-κB activation [64], [65]. The results of several *in vitro* studies have suggested that vitamin D may suppress the levels of inflammatory molecules that are induced via TLR signaling in multiple cell types [66], [67], [68], [69]. In addition, vitamin D was found to decrease NF-κB-induced inflammatory mediators in airway epithelial cells infected with RSV [27]. In contrast, additional reports have suggested inflammatory molecules induced in response to TLR agonists are not suppressed [7], [9], [10], [11]. Data from our experimental RSV infection model indicates that in lungs of calves with higher circulating 25(OH)D_3_, IL-12p40 was significantly upregulated compared to that in lungs of calves with lower circulating 25(OH)D_3_. Furthermore, we show that IL-8 and IFN-γ, were not inhibited in calves with high circulating 25(OH)D_3_ compared to calves with low 25(OH)D_3_. Taken together, our data indicate vitamin D did not suppress cytokines that can be induced via PRR ligation in response to RSV infection.

Cytokine secretion and immune responses are tightly controlled by an intricate balance between positive and negative regulatory signals that are delivered following an antigenic encounter. The recently described SOCS proteins act in a classic negative feedback loop to inhibit cytokine signaling pathways [70]. It is known that cytokines can, in fact, induce the expression of SOCS proteins. Moreover, a previous *in vitro* study has shown that RSV can modulate the expression of SOCS1 and SOCS3 [42]. We provide the first *in vivo* evidence that RSV infection induced significantly higher levels of SOC1 and SOCS3 expression in the inflamed lung. However, the two vitamin D treatment groups did not differ significantly in the levels of SOCS1 or SOCS3 gene expression. These data are indicative of a compensatory host tissue mechanism for regulating inflammatory responses that may be independent of vitamin D levels.

In conclusion, the major findings of the present study are that RSV activates the vitamin D intracrine pathway in the lesioned lung, and while vitamin D status does have an immunomodulatory role, there was no significant impact on the pathogenesis during the early phases of RSV infection. Examination of the potential effects of vitamin D status on RSV disease resolution will require additional longer term studies in calves with immunologically sufficient and deficient circulating 25(OH)D_3_ levels.

## Materials and Methods

### Calves

In two experiments, sixteen neonatal Holstein calves (n = 8/experiment) were randomly assigned to milk replacer diets with differing levels of vitamin D that resulted in two groups of calves (n = 4/group/experiment) which had high or low levels of circulating 25(OH)D_3_. Prior to this study, calves were healthy and were not vaccinated against bovine RSV. Calves were maintained on liquid milk replacer diets (Animix, LLC, Juneau, WI) containing 1700 IU of vitamin D/kg or 17,900 IU of vitamin D/kg for ten weeks prior to experimental challenge. A typical milk replacer diet would contain 11,000 IU/kg. The National Animal Disease Center Institutional Animal Care and Use Committee approved all animal procedures conducted in this study (ACUP #ARS-3993).

### Serum chemistries

Blood samples were collected from each calf on the day of experimental challenge and just prior to euthanasia. Serum 25(OH)D_3_ was quantified by radioimmunoassay as previously described [71]. Intra- and interassay coefficients of variation were 9.4% and 16.3%, respectively.

Serum calcium concentrations were determined by atomic absorption spectrometry (Perkin-Elmer, Norwalk, CT) using the method of Cali et al. [72]. Briefly, serum samples were prepared and measured in duplicate by diluting 100 µl of sample in 5 ml of 0.1% lanthanum oxide solution. A combined Ca^+2^ and Mg^+2^ lamp was used such that serum Mg^+2^ concentration was also determined. Plasma Ca^+2^ and Mg^+2^ concentrations were determined at 422.7 and 185.3 nm, respectively.

Serum phosphorus was measured colormetrically using procedures modified from Parekh and Jung [73], as validated in our laboratory. Briefly, serum (125 µl) was precipitated by acidification with 1 ml of molybdic-trichloroacetic acid solution. After mixing and equilibration for 5 min, this solution was centrifuged (1000× *g*) and 100 µl of supernatant was transferred in duplicate to a 96-well microtiter plate (Costar Corning, Acton, MA). Finally, 150 µl of *p*-phenylenediamine reagent (prepared by dissolving 1 g of *p*-phenylenediamine dihydrochloride in 100 ml of 5% Na_2_S_2_O_5_ solution) was added to all wells and the plate was incubated for 20 min at room temperature. Serum phosphorus was determined at a wavelength between 690 and 560 nm using a Thermo Max tunable microplate reader spectrophotometer (Molecular Devices, Sunnyvale, CA).

### RSV challenge model

Bovine RSV strain 375 used for inoculation of calves has been described in our previous studies [36], [43]. The inoculum was prepared from virus stock re-isolated from the lung of an infected animal and passaged less than 4 times on bovine turbinate cells. When 90% of virus-induced cytopathic effect was visible, flasks were frozen, and thawed twice. Media were pooled and centrifuged to remove cellular debris. Supernatants were filtered, aliquoted, and stored at −80°C. One aliquot was used to determine the tissue culture infective dose (TCID_50_/ml) by standard plaque assay.

The bovine RSV aerosol challenge model used was similar to that previously described by Woolums et al. [44]. Briefly, the challenge inoculum was delivered by nebulization into a mask covering the nostrils and mouth. The nebulization apparatus consisted of a compressed air tank, a jet nebulizer, and a mask (Trudell Medical International, London, Ontario, Canada). A rubber gasket was added to the mask, which sealed it securely to the muzzle. Compressed air (25 lb/in^2^) was used to jet nebulize the challenge inoculum directly into a holding reservoir. Upon inspiration, the nebulized inoculum was inhaled through a one-way valve into the mask and directly into the nostrils. Each calf received a 5 ml challenge inoculum containing approximately 10^4^ TCID_50_/ml of bovine RSV strain 375 during the nebulization period of 10–15 min.

### Clinical evaluation

Calves were examined daily for clinical signs. Rectal temperature, respiratory rate, evidence of ocular or nasal discharge, cough, dyspnea, and appetite were evaluated and recorded for each calf.

### Pathological evaluation

Calves were euthanized at day 7 post-inoculation using IV sodium pentobarbital. The thorax was opened and the lungs evaluated for gross lesions. The lungs were then removed from the thorax for subsequent tissue collection. Sections of lung were collected for histopathological evaluation. Tissues were fixed by immersion in 10% neutral buffered formalin for 24 h and transferred to 90% ethanol. Samples were processed by routine paraffin-embedment processing techniques, 5 µm sections were cut and stained with hematoxylin and eosin. Microscopic lesions were evaluated by a pathologist without knowledge of vitamin D treatment group assignment. The severity of microscopic lesions was evaluated by examining sections to confirm the presence of any or all of the following changes: 1) atelectasis; 2) alveolar septal expansion due to cellular infiltrates; 3) pulmonary edema, evidenced by expansion of interlobular septae; 4) hyperplasia of bronchial associated lymphoid tissue (BALT) characterized by cuffing of bronchi with lymphoid follicles; 5) intra-alveolar and intra-bronchiolar infiltrates of neutrophils, macrophages or lymphocytes 6) attenuation of airway (bronchi and bronchioles) epithelium due to necrosis and loss of epithelial cells; and 7) presence of syncytia. Most severely affected sections were characterized by the presence of all 7 lesions (score = 7), while the least severely affected sections contained only one of the lesions (score = 1).

### Virus Isolation

Nasal swabs and samples of snap-frozen lung that were collected from each calf at necropsy were processed for virus isolation as follows. Nasal swabs placed in PBS were vortexed, samples clarified by centrifugation (800× *g*, 15 min, 4°C), supernatants harvested, and stored at −80°C. Frozen lung tissue was disrupted by grinding with a mortar and pestle. A 10% suspension of the lung homogenate was made in minimal essential medium (MEM). The suspension was clarified by centrifugation (1000× *g*, 30 min, 4°C), the supernatant harvested and stored at −80°C. Supernatants from nasal swabs or lung homogenates were inoculated onto confluent monolayers of Madin-Darby Bovine Kidney cells and incubated for 90 min at 37°C with 5% CO_2_. After incubation, the inoculum was aspirated and fresh supplemented medium was added. Cultures were incubated at 37°C, 5%CO_2_ and daily observations for cytopathic effect were conducted and recorded for 7 days following inoculation of cells.

### Real-time PCR

Lung samples from a representative gross lesion and non-lesioned tissue from each calf were collected and stored in RNA*later*® (Invitrogen, Life Technologies, Carlsbad, CA). RNA was isolated from lung tissue samples using the Trizol Reagent (Invitrogen, Life Technologies) according to manufacturer's instructions. In addition, RNA was obtained from lung sections of 4 non-infected, control calves. The RNA concentration in each sample was determined using a NanoDrop 2000 spectrophotometer (Thermo Scientific, Wilmington, DE). RNA (300 ng per sample) was DNase-treated and cDNA synthesized using random primers according to the manufacturer's instructions (Invitrogen, Life Technologies). SYBR Green-based real-time PCR was performed on a 7300 Real-Time PCR System (Applied Biosystems, Life Technologies, Carlsbad, CA). The following amplification conditions were used: 95°C for 10 min, followed by 40 cycles at 95°C for 15 s and 60°C for 1 min, and a final dissociation step. Each reaction contained 10 µl SYBR Green master mix (Applied Biosystems), 1.25 µl each of 10 µM forward and reverse primers (Table 1), 5.5 µl dH_2_0, and 2 µl of cDNA. Relative gene expression was determined using the 2^−ΔΔCt^ method [74]. RPS9 was used as the reference gene as previously described [6], [7].

**Table 1:** Primers used in the present study.

Gene (alternate name)	Accession number^1^	Strand	Sequence (5′-3′)^2^
IL-1β	X54796	ForwardReverse	ATGGGTGTTCTGCATGAG AAGGCCACAGGAATCTTG
IL-4	NM_173921.2	ForwardReverse	GCGGACTTGACAGGAATCTC GCGTACTTGTGCTCGTG
IL-6	NM_173923.2	ForwardReverse	CTGAAGCAAAAGATCGCAGATCTA CTCGTTTGAAGACTGCATCTTCTC
IL-8	X78306	ForwardReverse	AAGCTGGCTGTTGCTCTC GGCATCAGAAGTTCTGTACTC
IL-10	NM_174088.1	ForwardReverse	TTACCTGGAGGAGGTGATG GTTCACGTGCTCCTTGATG
IL-12p40	AF004024	ForwardReverse	AAGTCACATGCCACAAGG CACTCCAGAATGAGCTGTAG
IFN-γ	NM_174088.1	ForwardReverse	AGAATCTCTTTCGAGGCCGGAG TATTGCAGGCAGGAGGACCATTAC
TGF-β	NM_001166068.1	ForwardReverse	CTGAGCCAGAGGCGGACTAC TGCCGTATTCCACCATTAGCA
TNF-α	NM_173966.2	ForwardReverse	CGGGGTAATCGGCCCCCAGA GGCAGCCTTGGCCCCTGAAG
SOCS1	CB460055	ForwardReverse	CACAGCAGAAAAATAAAGCCAGAGA CTCGTACCTCCTACCTCTTCATGTT
SOCS3	NM_174466	ForwardReverse	GGCCACTCTCCAACATCTCTGT TCCAGGAACTCCCGAATGG
VDR	NM_001167932	ForwardReverse	AGCCACCGGCTTCCATTTCA AACAGCGCCTTCCGCTTCAT
1α-OHase (CYP27B1)	NM_001192284	ForwardReverse	TGGACCAGATGTTTTGCATTCGC TTCTAGACTGGTTCCTCATGGCT
24-OHase (CYP24A1)	NM_001191417	ForwardReverse	GAAGACTGGCAGAGGGTCAG CAGCCAAGACCTCGTTGATT
RPS9	NM_001101152	ForwardReverse	CGCCTCGACCAAGAGCTGAAG CTCCAGACCTCACGTTTGTTCC

1 Accession numbers from NCBI database http://www.ncbi.nlm.nih.gov.

2 Primer sequences have been published previously [6], [7], [36], [37] or were designed in our laboratory.

### Statistical analysis

Body temperatures were analyzed using a repeated measures ANOVA (Prism, GraphPad, La Jolla, CA). Comparisons of serum 25(OH)D_3_ levels in the high and low vitamin D groups were conducted using a two-tailed Student's t test (Prism, GraphPad). ΔΔCt values were used in the statistical analyses of relative gene expression. Analyses to compare high versus low vitamin D treatment groups or lesioned versus non-lesioned lung samples were performed using a two-tailed Student's t-test statistic (Prism, GraphPad). ΔΔCt values ± SE were transformed (2^−ΔΔCt^) and are shown as the expression relative to control lung samples [74].

## References

[1] Jones G, Strugnell SA, DeLuca HF (1998). Current understanding of the molecular actions of vitamin D.. Physiol Rev.

[2] Adams JS, Liu PT, Chun R, Modlin RL, Hewison M (2007). Vitamin D in defense of the human immune response.. Ann N Y Acad Sci.

[3] Mora JR, Iwata M, von Andrian UH (2008). Vitamin effects on the immune system: vitamins A and D take centre stage.. Nat Rev Immunol.

[4] Hewison M (2011). Vitamin D and innate and adaptive immunity.. Vitam Horm.

[5] Bikle DD (2011). Vitamin D regulation of immune function.. Vitam Horm.

[6] Nelson CD, Reinhardt TA, Beitz DC, Lippolis JD (2010). In vivo activation of the intracrine vitamin D pathway in innate immune cells and mammary tissue during a bacterial infection.. PloS One.

[7] Nelson CD, Reinhardt TA, Thacker TC, Beitz DC, Lippolis JD (2010). Modulation of the bovine innate immune response by production of 1alpha,25-dihydroxyvitamin D(3) in bovine monocytes.. J Dairy Sci.

[8] White JH (2008). Vitamin D signaling, infectious diseases, and regulation of innate immunity.. Infect Immun.

[9] Fagan DL, Prehn JL, Adams JS, Jordan SC (1991). The human myelomonocytic cell line U-937 as a model for studying alterations in steroid-induced monokine gene expression: marked enhancement of lipopolysaccharide-stimulated interleukin-1 beta messenger RNA levels by 1,25-dihydroxyvitamin D3.. Mol Endocrinol.

[10] Prehn JL, Fagan DL, Jordan SC, Adams JS (1992). Potentiation of lipopolysaccharide-induced tumor necrosis factor-alpha expression by 1,25-dihydroxyvitamin D3.. Blood.

[11] Bhalla AK, Amento EP, Krane SM (1986). Differential effects of 1,25-dihydroxyvitamin D3 on human lymphocytes and monocyte/macrophages: inhibition of interleukin-2 and augmentation of interleukin-1 production.. Cell Immunol.

[12] Khoo AL, Chai LY, Koenen HJ, Oosting M, Steinmeyer A (2011). Vitamin D(3) down-regulates proinflammatory cytokine response to Mycobacterium tuberculosis through pattern recognition receptors while inducing protective cathelicidin production.. Cytokine.

[13] Vidyarani M, Selvaraj P, Jawahar MS, Narayanan PR (2007). 1, 25 Dihydroxyvitamin D3 modulated cytokine response in pulmonary tuberculosis.. Cytokine.

[14] Cippitelli M, Santoni A (1998). Vitamin D3: a transcriptional modulator of the interferon-gamma gene.. Eur J Immunol.

[15] Saggese G, Federico G, Balestri M, Toniolo A (1989). Calcitriol inhibits the PHA-induced production of IL-2 and IFN-gamma and the proliferation of human peripheral blood leukocytes while enhancing the surface expression of HLA class II molecules.. J Endocrinol Invest.

[16] Reichel H, Koeffler HP, Tobler A, Norman AW (1987). 1 alpha,25-Dihydroxyvitamin D3 inhibits gamma-interferon synthesis by normal human peripheral blood lymphocytes.. Proc Natl Acad Sci USA.

[17] Bryce J, Boschi-Pinto C, Shibuya K, Black RE (2005). WHO estimates of the causes of death in children.. Lancet.

[18] Williams B, Williams AJ, Anderson ST (2008). Vitamin D deficiency and insufficiency in children with tuberculosis.. Pediatr Infect Dis J.

[19] Wayse V, Yousafzai A, Mogale K, Filteau S (2004). Association of subclinical vitamin D deficiency with severe acute lower respiratory infection in Indian children under 5 y.. Eur J Clin Nutr.

[20] Karatekin G, Kaya A, Salihoglu O, Balci H, Nuhoglu A (2009). Association of subclinical vitamin D deficiency in newborns with acute lower respiratory infection and their mothers.. Eur J Clin Nutr.

[21] Grant CC, Wall CR, Gibbons MJ, Morton SM, Santosham M (2011). Child nutrition and lower respiratory tract disease burden in New Zealand: a global context for a national perspective.. J Paediatr Child Health.

[22] Stensballe LG, Devasundaram JK, Simoes EA (2003). Respiratory syncytial virus epidemics: the ups and downs of a seasonal virus.. Pediatr Infect Dis J.

[23] Walker VP, Modlin RL (2009). The vitamin D connection to pediatric infections and immune function.. Pediatr Res.

[24] Rook GA, Steele J, Fraher L, Barker S, Karmali R (1986). Vitamin D3, gamma interferon, and control of proliferation of Mycobacterium tuberculosis by human monocytes.. Immunology.

[25] Rook G (1986). Vitamin D and tuberculosis.. Tubercle.

[26] Liu PT, Stenger S, Li H, Wenzel L, Tan BH (2006). Toll-like receptor triggering of a vitamin D-mediated human antimicrobial response.. Science.

[27] Hansdottir S, Monick MM, Lovan N, Powers L, Gerke A (2010). Vitamin D decreases respiratory syncytial virus induction of NF-kappaB-linked chemokines and cytokines in airway epithelium while maintaining the antiviral state.. J Immunol.

[28] Wejse C, Gomes VF, Rabna P, Gustafson P, Aaby P (2009). Vitamin D as supplementary treatment for tuberculosis: a double-blind, randomized, placebo-controlled trial.. Am J Respir Crit Care Med.

[29] Martineau AR, Wilkinson RJ, Wilkinson KA, Newton SM, Kampmann B (2007). A single dose of vitamin D enhances immunity to mycobacteria.. Am J Respir Crit Care Med.

[30] Bruce D, Ooi JH, Yu S, Cantorna MT (2010). Vitamin D and host resistance to infection? Putting the cart in front of the horse.. Exp Biol Med (Maywood).

[31] Lippolis JD, Reinhardt TA, Sacco RA, Nonnecke BJ, Nelson CD (2011). Treatment of an intramammary bacterial infection with 25-hydroxyvitamin D(3).. PloS One.

[32] Becker S, Quay J, Soukup J (1991). Cytokine (tumor necrosis factor, IL-6, and IL-8) production by respiratory syncytial virus-infected human alveolar macrophages.. J Immunol.

[33] Takeuchi R, Tsutsumi H, Osaki M, Haseyama K, Mizue N (1998). Respiratory syncytial virus infection of human alveolar epithelial cells enhances interferon regulatory factor 1 and interleukin-1beta-converting enzyme gene expression but does not cause apoptosis.. J Virol.

[34] Takeuchi R, Tsutsumi H, Osaki M, Sone S, Imai S (1998). Respiratory syncytial virus infection of neonatal monocytes stimulates synthesis of interferon regulatory factor 1 and interleukin-1beta (IL-1beta)-converting enzyme and secretion of IL-1beta.. J Virol.

[35] Zhang Y, Luxon BA, Casola A, Garofalo RP, Jamaluddin M (2001). Expression of respiratory syncytial virus-induced chemokine gene networks in lower airway epithelial cells revealed by cDNA microarrays.. J Virol.

[36] Fach SJ, Meyerholz DK, Gallup JM, Ackermann MR, Lehmkuhl HD (2007). Neonatal ovine pulmonary dendritic cells support bovine respiratory syncytial virus replication with enhanced interleukin (IL)-4 and IL-10 gene transcripts.. Viral Immunol.

[37] Fach SJ, Olivier A, Gallup JM, Waters TE, Ackermann MR (2010). Differential expression of cytokine transcripts in neonatal and adult ovine alveolar macrophages in response to respiratory syncytial virus or toll-like receptor ligation.. Vet Immunol Immunopathol.

[38] D'Ambrosio D, Cippitelli M, Cocciolo MG, Mazzeo D, Di Lucia P (1998). Inhibition of IL-12 production by 1,25-dihydroxyvitamin D3. Involvement of NF-kappaB downregulation in transcriptional repression of the p40 gene.. J Clin Invest.

[39] Cohen-Lahav M, Douvdevani A, Chaimovitz C, Shany S (2007). The anti-inflammatory activity of 1,25-dihydroxyvitamin D3 in macrophages.. J Steroid Biochem Mol Biol.

[40] Harant H, Andrew PJ, Reddy GS, Foglar E, Lindley IJ (1997). 1alpha,25-dihydroxyvitamin D3 and a variety of its natural metabolites transcriptionally repress nuclear-factor-kappaB-mediated interleukin-8 gene expression.. Eur J Biochem.

[41] Rubtsov YP, Rasmussen JP, Chi EY, Fontenot J, Castelli L (2008). Regulatory T cell-derived interleukin-10 limits inflammation at environmental interfaces.. Immunity.

[42] Moore EC, Barber J, Tripp RA (2008). Respiratory syncytial virus (RSV) attachment and nonstructural proteins modify the type I interferon response associated with suppressor of cytokine signaling (SOCS) proteins and IFN-stimulated gene-15 (ISG15).. Virology J.

[43] Meyerholz DK, Grubor B, Fach SJ, Sacco RE, Lehmkuhl HD (2004). Reduced clearance of respiratory syncytial virus infection in a preterm lamb model.. Microbes Infect.

[44] Woolums AR, Anderson ML, Gunther RA, Schelegle ES, LaRochelle DR (1999). Evaluation of severe disease induced by aerosol inoculation of calves with bovine respiratory syncytial virus.. Am J Vet Res.

[45] Lapin CD, Hiatt PW, Langston C, Mason E, Piedra PT (1993). A lamb model for human respiratory syncytial virus infection.. Pediatr Pulmonol.

[46] Larsen LE (2000). Bovine respiratory syncytial virus (BRSV): a review.. Acta Vet Scand.

[47] Neilson KA, Yunis EJ (1990). Demonstration of respiratory syncytial virus in an autopsy series.. Pediatr Pathol.

[48] Castleman WL, Lay JC (1990). Morphometric and ultrastructural study of postnatal lung growth and development in calves.. Am J Vet Res.

[49] Flecknoe SJ, Wallace MJ, Cock ML, Harding R, Hooper SB (2003). Changes in alveolar epithelial cell proportions during fetal and postnatal development in sheep.. Am J Physiol Lung Cell Mol Physiol.

[50] Bruce D, Whitcomb JP, August A, McDowell MA, Cantorna MT (2009). Elevated non-specific immunity and normal Listeria clearance in young and old vitamin D receptor knockout mice.. Int Immunol.

[51] Ehrchen J, Helming L, Varga G, Pasche B, Loser K (2007). Vitamin D receptor signaling contributes to susceptibility to infection with Leishmania major.. FASEB J.

[52] Waters WR, Palmer MV, Nonnecke BJ, Whipple DL, Horst RL (2004). Mycobacterium bovis infection of vitamin D-deficient NOS2−/− mice.. Microb Pathog.

[53] Cantorna MT, Hullett DA, Redaelli C, Brandt CR, Humpal-Winter J (1998). 1,25-Dihydroxyvitamin D3 prolongs graft survival without compromising host resistance to infection or bone mineral density.. Transplantation.

[54] Hansdottir S, Monick MM, Hinde SL, Lovan N, Look DC (2008). Respiratory epithelial cells convert inactive vitamin D to its active form: potential effects on host defense.. J Immunol.

[55] Gyetko MR, Hsu CH, Wilkinson CC, Patel S, Young E (1993). Monocyte 1 alpha-hydroxylase regulation: induction by inflammatory cytokines and suppression by dexamethasone and uremia toxin.. J Leukocyte Biol.

[56] Stoffels K, Overbergh L, Bouillon R, Mathieu C (2007). Immune regulation of 1alpha-hydroxylase in murine peritoneal macrophages: unravelling the IFNgamma pathway.. J Steroid Biochem Mol Biol.

[57] Pike JW, Meyer MB, Bishop KA (2011). Regulation of target gene expression by the vitamin D receptor - an update on mechanisms.. Rev Endocr Metab Dis.

[58] Holick MF (2007). Vitamin D deficiency.. N Engl J Med.

[59] McDermott CM, Beitz DC, Littledike ET, Horst RL (1985). Effects of dietary vitamin D3 on concentrations of vitamin D and its metabolites in blood plasma and milk of dairy cows.. J Dairy Sci.

[60] Abu-Harb M, Bell F, Finn A, Rao WH, Nixon L (1999). IL-8 and neutrophil elastase levels in the respiratory tract of infants with RSV bronchiolitis.. Eur Respir J.

[61] Noah TL, Henderson FW, Wortman IA, Devlin RB, Handy J (1995). Nasal cytokine production in viral acute upper respiratory infection of childhood.. J Infect Dis.

[62] Zlotnik A, Yoshie O (2000). Chemokines: a new classification system and their role in immunity.. Immunity.

[63] Sha Q, Truong-Tran AQ, Plitt JR, Beck LA, Schleimer RP (2004). Activation of airway epithelial cells by toll-like receptor agonists.. Am J Respir Cell Mol Biol.

[64] Rudd BD, Burstein E, Duckett CS, Li X, Lukacs NW (2005). Differential role for TLR3 in respiratory syncytial virus-induced chemokine expression.. J Virol.

[65] Liu P, Jamaluddin M, Li K, Garofalo RP, Casola A (2007). Retinoic acid-inducible gene I mediates early antiviral response and Toll-like receptor 3 expression in respiratory syncytial virus-infected airway epithelial cells.. J Virol.

[66] Cohen ML, Douvdevani A, Chaimovitz C, Shany S (2001). Regulation of TNF-alpha by 1alpha,25-dihydroxyvitamin D3 in human macrophages from CAPD patients.. Kidney Int.

[67] Penna G, Adorini L (2000). 1 alpha,25-dihydroxyvitamin D3 inhibits differentiation, maturation, activation, and survival of dendritic cells leading to impaired alloreactive T cell activation.. J Immunol.

[68] Takahashi K, Nakayama Y, Horiuchi H, Ohta T, Komoriya K (2002). Human neutrophils express messenger RNA of vitamin D receptor and respond to 1alpha,25-dihydroxyvitamin D3.. Immunopharmacol Immunotoxicol.

[69] Sadeghi K, Wessner B, Laggner U, Ploder M, Tamandl D (2006). Vitamin D3 down-regulates monocyte TLR expression and triggers hyporesponsiveness to pathogen-associated molecular patterns.. Eur J Immunol.

[70] Yoshimura A, Naka T, Kubo M (2007). SOCS proteins, cytokine signalling and immune regulation.. Nat Rev Immunol.

[71] Hollis BW, Kamerud JQ, Selvaag SR, Lorenz JD, Napoli JL (1993). Determination of vitamin D status by radioimmunoassay with an 125I-labeled tracer.. Clin Chem.

[72] Cali JP, Bowers GN, Young DS (1973). A referee method for the determination of total calcium in serum.. Clin Chem.

[73] Parekh AC, Jung DH (1970). Serum inorganic phosphorus determination using p-phenylenediamine as a reducing agent.. Clin Chim Acta.

[74] Livak KJ, Schmittgen TD (2001). Analysis of relative gene expression data using real-time quantitative PCR and the 2(−Delta Delta C(T)) method.. Methods.

